# Efficacy of third-line chemotherapy following nanoliposomal irinotecan combined with fluorouracil and folinic acid as second-line treatment for unresectable pancreatic cancer

**DOI:** 10.3389/fonc.2025.1626689

**Published:** 2025-07-17

**Authors:** Keisuke Miwa, Reina Kawasaki, Mototsugu Shimokawa, Taiga Otsuka, Toshimitsu Tanaka, Masaru Fukahori, Taro Shibuki, Junichi Nakazawa, Shiho Arima, Futa Koga, Yujiro Ueda, Yoshihito Kubotsu, Hozumi Shimokawa, Shigeyuki Takeshita, Kazuo Nishikawa, Azusa Komori, Satoshi Otsu, Ayumu Hosokawa, Tatsunori Sakai, Hisanobu Oda, Machiko Kawahira, Shuji Arita, Takuya Honda, Hiroki Taguchi, Kengo Tsuneyoshi, Toshihiro Fujita, Takahiro Sakae, Yasunori Kawaguchi, Tsuyoshi Shirakawa, Toshihiko Mizuta, Kenji Mitsugi

**Affiliations:** ^1^ Multidisciplinary Treatment Cancer Center, Kurume University Hospital, Fukuoka, Japan; ^2^ Division of Integrative Medical Oncology, Saiseikai Kumamoto Hospital, Kumamoto, Japan; ^3^ Clinical Research Institute, National Kyushu Cancer Center, Fukuoka, Japan; ^4^ Department of Biostatistics, Yamaguchi University Graduate School of Medicine, Yamaguchi, Japan; ^5^ Department of Internal Medicine, Minato Medical Clinic, Fukuoka, Japan; ^6^ Department for the Promotion of Drug and Diagnostic Development, Division of Drug and Diagnostic Development Promotion, Translational Research Support Office, National Cancer Center Hospital East, Chiba, Japan; ^7^ Department of Hepatobiliary and Pancreatic Oncology, National Cancer Center Hospital East, Chiba, Japan; ^8^ Department of Medical Oncology, Kagoshima City Hospital, Kagoshima, Japan; ^9^ Digestive and Lifestyle Diseases, Kagoshima University Graduate School of Medical and Dental Sciences, Kagoshima, Japan; ^10^ Department of Hepatobiliary and Pancreatology, Saga Medical Center Koseikan, Saga, Japan; ^11^ Department of Hematology and Oncology, Japanese Red Cross Kumamoto Hospital, Kumamoto, Japan; ^12^ Department of Internal Medicine, Karatsu Red Cross Hospital, Saga, Japan; ^13^ Department of Hematology Oncology, Japan Community Healthcare Organization Kyushu Hospital, Fukuoka, Japan; ^14^ Department of Medical Oncology, Hamanomachi Hospital, Fukuoka, Japan; ^15^ Department of Gastroenterology, Japanese Red Cross Nagasaki Genbaku Hospital, Nagasaki, Japan; ^16^ Department of Medical Oncology and Hematology, Oita University Faculty of Medicine, Oita, Japan; ^17^ Department of Gastrointestinal Medical Oncology, National Hospital Organization Shikoku Cancer Center, Ehime, Japan; ^18^ Department of Clinical Oncology, University of Miyazaki Hospital, Miyazaki, Japan; ^19^ Department of Medical Oncology, NHO Kumamoto Medical Center, Kumamoto, Japan; ^20^ Department of Gastroenterology, Kagoshima Kouseiren Hospital, Kagoshima, Japan; ^21^ Department of Chemotherapy, Miyazaki Prefectural Miyazaki Hospital, Miyazaki, Japan; ^22^ Department of Gastroenterology and Hepatology, Nagasaki University Graduate School of Biomedical Sciences, Nagasaki, Japan; ^23^ Department of Gastroenterology, Izumi General Medical Center, Kagoshima, Japan; ^24^ Department of Gastroenterology, Kagoshima City Hospital, Kagoshima, Japan; ^25^ Department of Gastroenterology, Saiseikai Sendai Hospital, Kagoshima, Japan; ^26^ Department of Gastroenterology, Asakura Medical Association Hospital, Fukuoka, Japan; ^27^ Clinical Hematology Oncology Treatment Study Group, Fukuoka, Japan; ^28^ Department of Medical Checkup Center, Eikoh Hospital, Fukuoka, Japan; ^29^ Department of Internal Medicine, Fujikawa Hospital, Saga, Japan; ^30^ Department of Medical Oncology, Sasebo Kyosai Hospital, Nagasaki, Japan

**Keywords:** pancreatic cancer, chemotherapy, third-line treatment, nanoliposomal irinotecan, best supportive care

## Abstract

**Introduction:**

The significance of third-line chemotherapy (CTx) in unresectable pancreatic cancer (UPC) remains unclear. This study evaluated the therapeutic impact of third-line CTx after nanoliposomal irinotecan and fluorouracil combined with folinic acid (nal-IRI + 5-FU/LV) therapy as second-line CTx for UPC.

**Methods:**

Between June 2020 and May 2021, 104 patients who received nal-IRI + 5-FU/LV therapy as second-line CTx were retrospectively analyzed for post-discontinuation survival (PDS) and overall survival (OS). Comparisons were made between patients transitioning to third-line CTx and those receiving best supportive care (BSC), using a Cox proportional hazards model adjusted for patient background.

**Results:**

Of the cohort, 34 patients received third-line CTx, whereas 61 transitioned to BSC. The median OS from first-line CTx in the third-line CTx group was 18.0 months, with a median OS of 9.7 months from second-line CTx. Adjusted median PDS following second-line CTx was 6.5 months for the third-line CTx group compared to 2.3 months for the BSC group (adjusted hazard ratio 0.16; 95% confidence interval 0.08–0.32; P < 0.01).

**Conclusion:**

Third-line CTx should be actively considered for patients with UPC, as the approach may significantly extend survival in those who can tolerate the treatment.

## 1 Introduction

Metastatic or recurrent unresectable pancreatic cancer (UPC) has a poor prognosis, with a 5-year survival rate of only 1.3% (1), making it an extremely poor prognostic disease with a life expectancy of less than 5 months at diagnosis (2). Pancreatic cancer-related mortality is increasing worldwide (3), with an estimated 40,000 new cases diagnosed and 33,000 deaths annually in Japan (4).

For the past two decades, gemcitabine-based chemotherapy (CTx) has served as the standard of care for patients with UPC (5, 6). Recently, combinations such as FOLFIRINOX (oxaliplatin, folinic acid, irinotecan, and fluorouracil) and gemcitabine plus nab-paclitaxel have shown efficacy (7–9), establishing them as first-line CTx in Japan. Additionally, phase III trials have demonstrated the effectiveness of nanoliposomal irinotecan combined with fluorouracil and folinic acid (NFF) as a second-line CTx for patients resistant to gemcitabine-based regimens (10). This approach has gained widespread use in Japan, with real-world data supporting the role of second-line CTx in prolonging survival for patients with UPC (11). The efficacy of NFF as second-line CTx in Japan has already been reported (12).

Despite advancements in first-line and second-line therapies, randomized trials evaluating third-line CTx remain unavailable, leaving its clinical significance uncertain. In practice, the decision to pursue additional lines of CTx often depends on the patient’s general condition and preferences. Reports indicate that among patients receiving primary chemotherapy for UPC, 57% undergo second-line CTx, whereas 22% proceed to third-line CTx (13). A recent retrospective study reported a median progression-free survival (PFS) of 4.4 months and a median overall survival (OS) of 6.9 months for third-line CTx in patients with UPC (14), suggesting that third-line CTx may improve survival outcomes. However, data regarding the prognosis associated with later lines of CTx remain limited. Therefore, we conducted a retrospective study to evaluate the outcomes of patients resistant to NFF as second-line CTx for UPC, examining the potential benefits of third-line CTx.

## 2 Materials and methods

### 2.1 Patients

This multicenter, retrospective study of patients treated with NFF therapy as second-line or later-line CTx for unresectable or recurrent pancreatic cancer (NAPOLEON-2 Study) was conducted at 20 centers with oncology and gastroenterology specialists in Japan (12). We reviewed consecutive charts of patients with UPC who received NFF therapy as second-line or later-line CTx between June 2020 and May 2021, focusing on those who received NFF therapy as second-line treatment and subsequently transitioned to either third-line CTx or best supportive care (BSC). The NAPOLEON-2 study was approved by the Institutional Review Board of each participating institution and adhered to the principles of the Declaration of Helsinki. As this was a retrospective observational study conducted in Japan, informed consent was obtained using the opt-in/opt-out approach according to each participating institution’s policy.

NFF therapy consisted of a 90-min intravenous infusion of nanoliposomal irinotecan (70 mg/m^2^), a 46-h continuous intravenous infusion of fluorouracil (2400 mg/m^2^), and a 2-h intravenous infusion of folinic acid (200 mg/m^2^) every 2 weeks. Physicians had the discretion to implement dose reductions at the initiation of treatment or to modify the dose during treatment in response to toxicity. Treatment was discontinued upon disease progression, the occurrence of unacceptable adverse events (AEs), or a patient request. In certain cases, continuation of the NFF treatment regimen after disease progression was permitted if deemed feasible by the treating physician.

### 2.2 Assessment

This was a pre-planned analysis of the NAPOLEON-2 study. The primary endpoint of the study was OS. Secondary endpoints included the proportion of patients achieving an objective response, disease control, and PFS. This study analyzed the OS across all treatment lines and post-discontinuation survival (PDS) after second-line NFF therapy. OS was defined as the duration from the start date of primary or second-line CTx to the date of death from any cause or the last follow-up examination, PDS was defined as the period from the discontinuation of second-line NFF therapy to death from any cause or the last follow-up examination.

Computed tomography or magnetic resonance imaging was used to evaluate antitumor response, which was graded according to the Response Evaluation Criteria in Solid Tumors (RECIST) version 1.1 (15). Response were categorized as complete response, partial response, stable disease, or progressive disease. The objective response rate (ORR) included complete and partial responses, whereas the disease control rate (DCR) encompassed complete and partial responses alongside stable disease as the best response.

### 2.3 Statistical analysis

OS and PDS were estimated using the Kaplan-Meier method, with survival probabilities compared between the third-line CTx and BSC groups using the log-rank test and Cox proportional hazards model. The hazard ratio (HR) was expressed with a 95% confidence interval (95%CI), and differences were considered significant at P < 0.05. Patient characteristics were compared using standardized mean differences. Risk factors influencing PDS were analyzed using Cox proportional hazards models at the discontinuation of NFF treatment. Covariates for the adjusted HR in comparing PDS between the third-line CTx and BSC groups were selected by clinicians according to the international consensus statement for unresectable pancreatic cancer (16). Statistical analyses were performed using R ver. 4.2.0 (R Foundation for Statistical Computing, Vienna, Austria).

## 3 Results

### 3.1 Patients’ characteristics

**Figure 1:**
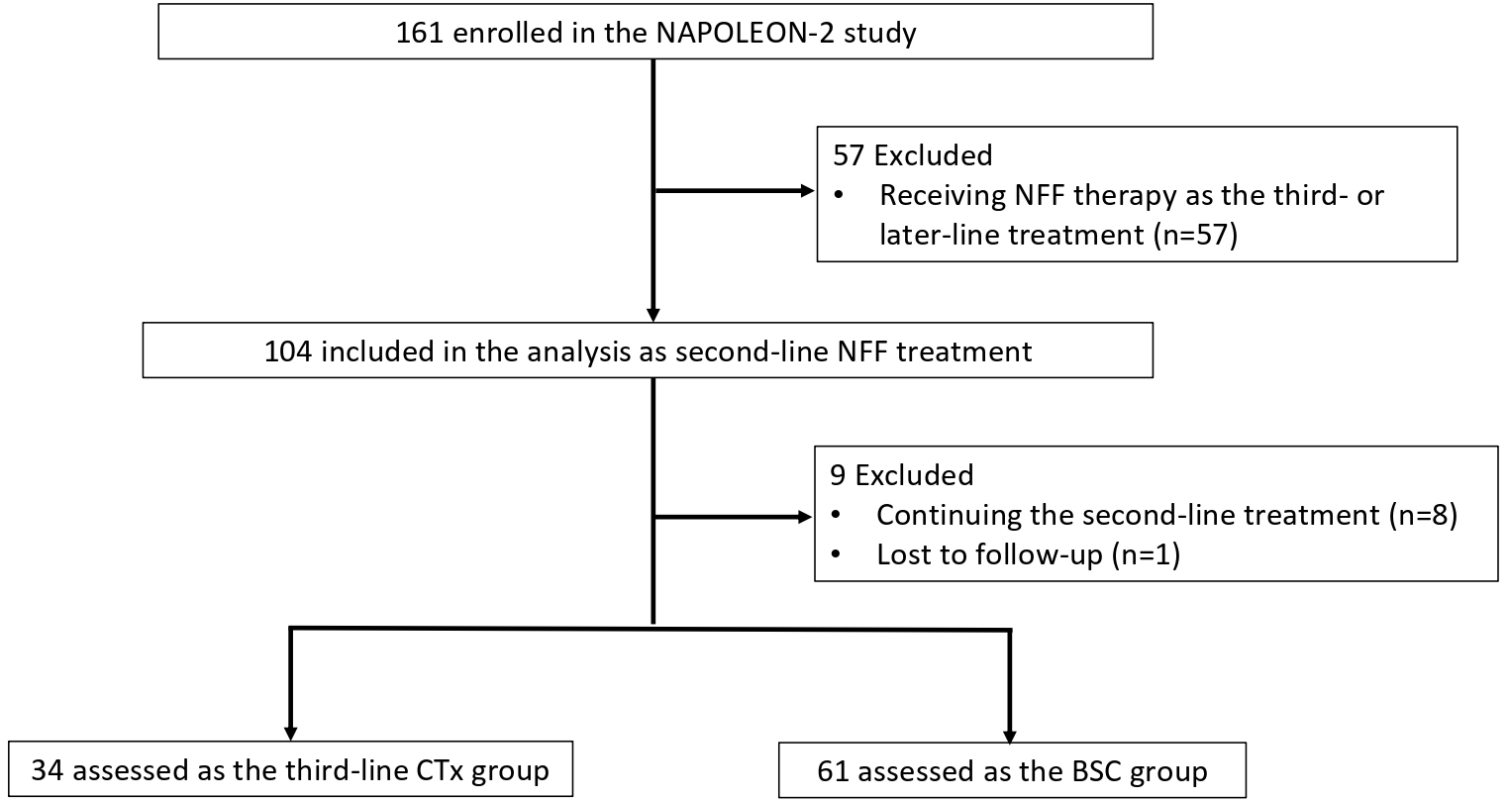
Flowchart of patient selection and progression. NFF, nanoliposomal irinotecan combined with fluorouracil and folinic acid; BSC, best supportive care; CTx, chemotherapy.

Between June 2020 and May 2021, 161 patients with UPC received NFF therapy across 20 institutions. After excluded 57 patients receiving NFF therapy as the third- or later-line treatment, 104 patients were included in this analysis as second-line CTx. Of these, 9 patients were excluded due to continuing NFF (n = 8) and lost to follow-up (n = 1). Finally, 34 and 61 patients were assessed as the third-line CTx group or BSC group, respectively (**Figure 1**). The median follow-up duration was 7.3 months (95% CI 5.6–8.9 months). **Table 1** shows the patient characteristics at the initiation of NFF therapy as a second-line treatment, and **Table 2** presents the patient characteristics at the end of NFF therapy. Both all of patients of third-line CTx group (n = 34) and BSC group (n =61) had distant metastasis. No significant differences in baseline characteristics were noted between the BSC and third-line CTx groups; however, the Eastern Cooperative Oncology Group performance status (ECOG PS) score and ORR were significantly better in the third-line CTx group than in the BSC group at the end of second-line CTx.

**Table 1:** Patient characteristics at the start of NFF therapy.

Characteristics		Best supportive care (n=61)	Third line treatment (n=34)	SMD
Age	Median (range)	70 (47–82)	65 (49–79)	0.21
Sex, n (%)	Men	38 (62)	18 (53)	0.19
ECOG PS, n (%)	0	25 (41)	19 (56)	0.48
	1	31 (51)	15 (44)	
	2, 3	5 (8)	0	
History of pancreatectomy, n (%)	Yes	9 (15)	4 (12)	0.09
History of biliary drainage, n (%)	Yes	14 (23)	6 (18)	0.13
Primary pancreatic site, n (%)	Head	18 (30)	15 (44)	0.31
Organization type, n (%)	Adenocarcinoma	54 (89)	32 (94)	0.20
	Other	4 (7)	1 (3)	
	Unknown	3 (5)	1 (3)	
Progression, n (%)	Distant metastasis cases	52 (85)	33 (97)	0.43
Distant metastasis site. n (%)	Liver	38 (62)	25 (74)	0.24
	Peritoneum	17 (28)	11 (32)	0.10
	Lung	10 (16)	3 (9)	0.23
Presence of ascites n (%)	Yes	12 (20)	4 (12)	0.22
Primary treatment n (%)	GEM + nab-PTX	60 (98)	33 (97)	0.31
	GEM + S-1	1 (2)	0	
	S-1	0	1 (3)	
Pre-treatment period, month	Median (range)	7.8 (1.8–33.0)	6.5 (1.4–24.0)	0.12
CA19-9 U/mL	Median (range)	1593 (0.6–489500)	711 (2–44320)	0.29
UGT1A1 gene polymorphism, n (%)	Homo, compound hetero	2 (3)	3 (9)	0.23

NFF, nanoliposomal irinotecan combined with fluorouracil and folinic acid; SMD, standardized mean difference; ECOG PS, Eastern Cooperative Oncology Group's performance status; GEM, gemcitabine; nab-PTX, nab-paclitaxel; CA19-9, carbohydrate antigen 19-9; UGT1A1, uridine diphosphate glucuronosyltransferase 1A1.

**Table 2:** Patient characteristics at the end of NFF therapy.

Characteristics		Best supportive care (n=61)	Third line treatment (n=34)	SMD
Progression, n (%)	Distant metastasis cases	61 (100)	34 (100)	0.00
Starting dose of nal-IRI, n (%)	No reduction	44 (72)	20 (59)	0.28
Starting dose of 5-FU, n (%)	No reduction	49 (80)	30 (88)	0.22
Relative dose intensity of nal-IRI	Median (range)	87.5 (55.5–105.7)	80.4 (58.8–102.0)	0.25
Relative dose intensity of 5-FU	Median (range)	93.9 (61.9–106.5)	96.5 (59.6–108.3)	0.02
NFF therapy course	Median (range)	4 (1–35)	5 (1–31)	0.23
Best tumor response, n (%)	PR	0	3 (9)	0.44
	PR + SD	29 (48)	13 (38)	0.19
Reasons for discontinuation of nal-IRI, n (%)	PD	54 (89)	33 (97)	0.36
	AE	5 (8)	1 (3)	
	Patient request	2 (3)	0	
ECOG PS, n (%)	0	8 (13)	9 (27)	1.22
	1	21 (34)	23 (68)	
	2	18 (30)	2 (6)	
	3	11 (18)	0	
	4	1 (2)	0	
	Unknown	2 (3)	0	
Age	Median (range)	71 (47–82)	66 (49–81)	0.21
CA19-9 U/mL	Median (range)	2618 (0.6–699800)	1961 (2.1–50115)	0.24

NFF, nanoliposomal irinotecan combined with fluorouracil and folinic acid; SMD, standardized mean difference; nal-IRI, nanoliposomal irinotecan; 5-FU, fluorouracil; ECOG PS, Eastern Cooperative Oncology Group's performance status; GEM, gemcitabine; nab-PTX, nab-paclitaxel; CA19-9, carbohydrate antigen 19-9; UGT1A1, uridine diphosphate glucuronosyltransferase 1A1.

### 3.2 Third-line regimens and tumor response

**Table 3** outlines the regimens employed in the third-line CTx group. The FOLFOX regimen was administered to 12 patients (35%), the modified FOLFIRINOX regimen to seven (21%), and S-1 alone to six (18%), accounting for more than half of the patients. Only one (3%) patient participated in the clinical trial. The anti-tumor effects of second-line NFF therapy in the third-line CTx and BSC groups are shown in **Table 4**. The third-line CTx group had a 9% response rate, whereas the BSC group had a 0% response rate, significantly higher than the 0% observed in the BSC group.

**Table 3:** Details of third-line chemotherapy regimen.

Regimens	Third-line CTx (n=34), n (%)
FOLFOX	12 (35)
mFOLFIRINOX	7 (21)
S-1	6 (18)
FOLFIRINOX	3 (9)
mFOLFOX6	2 (6)
GEM + S-1	2 (6)
FOLFIRI	1 (3)
Clinical trial	1 (3)

CTx, chemotherapy.

Regimen description

FOLFOX: 85 mg/m^2^ oxaliplatin, 100 mg/m^2^ folinic acid, followed by a 400 mg/m^2^ bolus of 5-FU and a 22-h 600 mg/m^2^ 5-FU infusion on day1, 100 mg/m^2^ folinic acid followed by a 400 mg/m^2^ bolus of 5-FU and a 22-h 600 mg/m^2^ 5-FU infusion on day 2, every 2 weeks. mFOLFIRINOX: 85 mg/m^2^ oxaliplatin, 150 mg/m^2^ irinotecan, and 200 mg/m^2^ folinic acid on day 1, followed by a 46-h infusion of 2400 mg/m2 5-FU every 2 weeks. S-1:40 mg/m^2^ once daily for 2 weeks, every 3 weeks. FOLFIRINOX: 85 mg/m^2^ oxaliplatin, 180mg/m^2^ irinotecan, 200 mg/m^2^ folinic acid on day 1, followed by a 400 mg/m^2^ bolus of 5-FU and a 46-h 2400 mg/m^2^ 5-FU infusion every 2 weeks. mFOLFOX6:85 mg/m^2^ oxaliplatin and 200 mg/m^2^ folinic acid on day 1, followed by a 400 mg/m^2^ bolus of 5-FU and a 46-h 2400 mg/m^2^ 5-FU infusion every 2 weeks. GEM+S-1:1000 mg/m^2^ gemcitabine on days 1, 8, and 40 mg/m^2^ once daily for 2 weeks and every 3 weeks. FOLFIRI: 150 mg/m^2^ irinotecan and 200 mg/m^2^ folinic acid on day 1, followed by a 400 mg/m^2^ bolus of 5-FU and a 46-h 2400 mg/m^2^ 5-FU infusion every 2 weeks.

**Table 4:** Anti-tumor effects of NFF therapy as a second-line treatment.

Objective response rate	BSC (n=61) n (%)	Third-line CTx (n=34) n (%)	SMD
CR	0 (0)	0 (0)	
PR	0 (0)	3 (9)	
SD	29 (48)	10 (29)	
PD	26 (43)	19 (56)	
NE	6 (10)	2 (6)	
RR (CR+PR))	0 (0)	3 (9)	0.44
DCR (CR+PR+SD)	29 (48)	13 (38)	0.19

NFF, nanoliposomal irinotecan combined with fluorouracil and folinic acid; CTx, chemotherapy; SMD, standardized mean difference; CR, complete response: PR, partial response; SD, stable disease; PD, progressive disease; NE, non-evaluable; RR, response rate; DCR, disease control rate.

### 3.3 Survival

**Figure 2:**
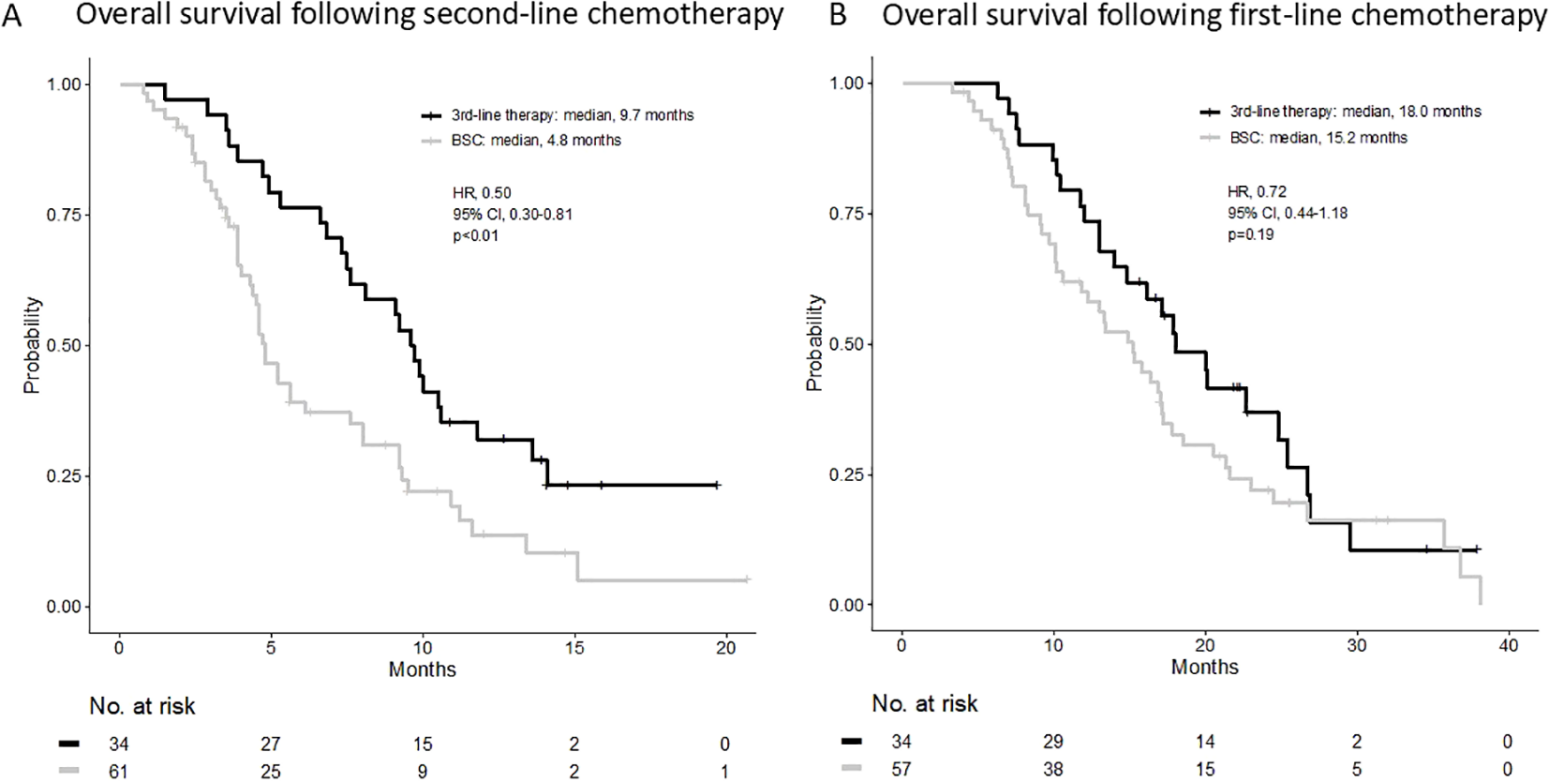
**(A)** Overall survival following second-line chemotherapy. **(B)** Overall survival following first-line chemotherapy. BSC, best supportive care; HR, hazard ratio; CI, confidence interval.

**Figure 3:**
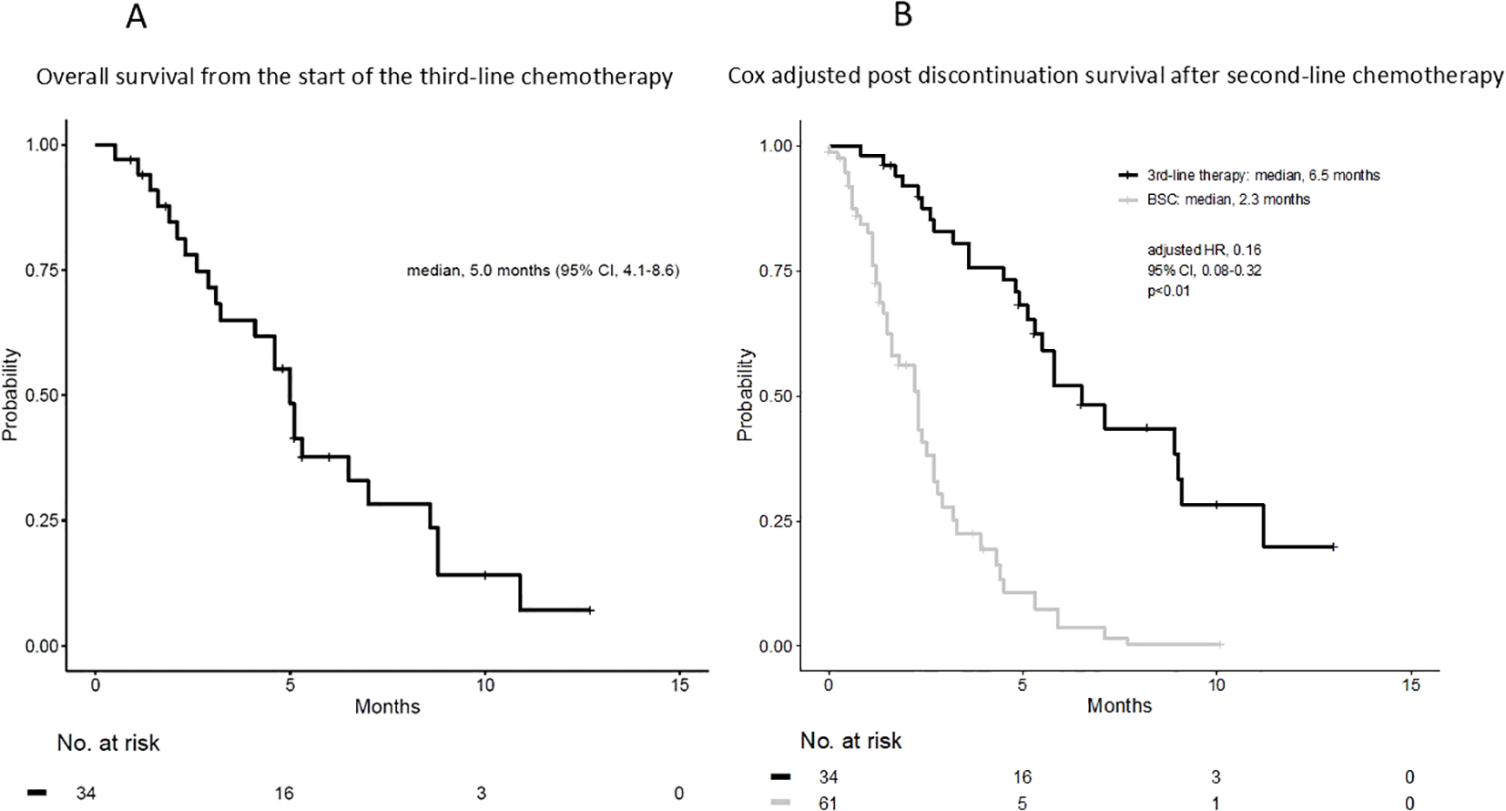
**(A)** Overall survival from the start of third-line chemotherapy. **(B)** Cox adjusted post-discontinuation survival after second-line chemotherapy. BSC, best supportive care; HR, hazard ratio; CI, confidence interval.

The median OS (mOS) from the start of second-line CTx was 9.7 and 4.8 months in the third-line CTx and BSC groups, respectively (HR 0.50; 95% CI 0.30–0.81; *P* < 0.01) (**Figure 2A**). The mOS from the start of first-line CTx was 18.0 and 15.2 months in the third-line CTx and BSC groups, respectively (HR 0.72; 95% CI 0.44–1.18; *P*=0.19) (**Figure 2B**). The mOS from the start of third-line CTx was 5.0 months (**Figure 3A**). The results of the univariate and multivariate analyses conducted to identify the determinants of PDS are listed in **Table 5**. Univariate analysis identified that ECOG PS score, absence of peritoneal metastases, and presence of third-line CTx were significantly associated with a longer PDS. Multivariate analysis identified age ≥75 years, absence of peritoneal metastases, and presence of third-line CTx as independent determinants of PDS. The Cox proportional hazards model-adjusted median PDS was 6.5 months for the third-line CTx group and 2.3 months for the BSC group (adjusted HR 0.16; 95% CI 0.08–0.32; *P* < 0.01) (**Figure 3B**).

**Table 5:** Cox regression analysis of post discontinuation survival after NFF therapy as second-line treatment.

Variables		Univariate			Multivariate		
		HR	95% CI	*p*	HR	95% CI	*p*
Age at the end of NFF therapy	<75 years old	1.75	0.94–3.26	0.08	3.55	1.72–7.32	<0.01
ECOG PS at the end of nal-IRI	0, 1	0.49	0.30–0.80	<0.01	0.87	0.50–1.53	0.63
Disease control (CR + PR + SD)	Yes	0.78	0.49–1.27	0.32	0.72	0.41–1.25	0.24
Liver metastasis	No	1.25	0.76–2.05	0.37	0.75	0.44–1.29	0.30
Peritoneal metastasis	No	0.51	0.31–0.85	0.01	0.49	0.29–0.83	<0.01
Third-line treatment	Yes	0.33	00.20–0.56	<0.01	0.16	0.08–0.32	<0.01

NFF, nanoliposomal irinotecan combined with fluorouracil and folinic acid; ECOG PS, Eastern Cooperative Oncology Group's performance status; nal-IRI, nanoliposomal irinotecan; CR, complete response; PR, partial response; SD, stable disease; HR, hazard ratio; CI, confidence interval.

## 4 Discussion

The prognosis for patients with UPC remains dismal (1, 2), with survival times often limited due to rapid disease progression and resistance to chemotherapy. Established first-line regimens for patients with UPC in good general condition are FOLFIRINOX, nanoliposomal irinotecan, fluorouracil, folinic acid and oxaliplatin (NALIRIFOX), and gemcitabine plus albumin-bound paclitaxel, all validated through key clinical trials (7–9, 17, 18). For second-line treatment, NFF has been widely recognized as the standard regimen for patients with metastatic pancreatic cancer (MPC) following gemcitabine-based first-line therapy (10). However, no standard regimen has been established for third-line CTx in patients with MPC, and the relevance of such interventions remains unclear. In this study, we investigated third-line treatment and its outcomes in patients with MPC who received NFF therapy as second-line CTx. The findings provide insights into the potential benefits and limitations of third-line CTx, highlighting areas for further investigation in this challenging clinical setting.

Several reports have documented outcomes of third-line CTx in patients with MPC, showing a median OS of 4.9–6.9 months (14, 19, 20). However, these studies have all been retrospective cohort studies, and no randomized controlled trials have evaluated third-line CTx in patients with MPC. To our knowledge, this is the first study to evaluate the therapeutic effects of third-line CTx where second-line CTx was uniformly limited to NFF therapy in patients with UPC. In this study, the median OS for third-line CTx was 5.0 months, aligning with previously reported findings. These results suggest that third-line CTx may contribute to prolonged OS when considered feasible and tolerated. Among all patients who received second-line CTx in this study, only 36% (34/95) transitioned to third-line CTx, with the majority being transferred to BSC. This data adds valuable insight into the real-world treatment landscape. Furthermore, 10 (29%) of the 34 patients who underwent third-line CTx proceeded to fourth-line CTx, potentially influencing OS outcomes in the third-line CTx group. Although baseline characteristics between the third-line CTx and BSC groups were comparable at the start of second-line NFF therapy, the ECOG PS was significantly better in the third-line CTx group at the end of second-line therapy. This difference likely guided physicians’ decisions to recommend third-line CTx. Interestingly, PFS during second-line CTx did not significantly differ between the third-line CTx and BSC groups (**Supplementary Figure S1**), indicating that the treatment effect of second-line CTx in patients with UPC does not reflect the rate of transition to third-line CTx.

In this study, comparing the PDS of the third-line CTx and BSC groups after third-line CTx, univariate analysis showed that ECOG PS 0–1, absence of peritoneal metastasis, and presence of third-line CTx were significantly associated with longer PDS. Multivariate analysis showed that age ≥75 years, absence of peritoneal metastases, and presence of third-line CTx were significantly associated with longer PDS. In both analyses, the presence or absence of peritoneal metastases and third-line CTx significantly affected PDS. In an analysis adjusted by patient background factors, the median PDS for the third-line CTx and BSC groups was 6.5 and 2.3 months, respectively, which we believe is valuable data to provide to patients with UPC. Furthermore, the median OS from first-line treatment in the third-line CTx group in this study was 18.0 months, which compares favorably with previous pivotal studies (7, 9, 17). This is presumably because of the establishment of second-line CTx for patients with UPC and the implementation of third-line CTx.

This study had several limitations. Firstly, significant differences in patient background factors existed between the third-line CTx and BSC groups, which may have influenced the physicians’ treatment decisions. As this research was an observational study in a real-world setting rather than a randomized study, the adjusted HR provided some information about the efficacy of third-line CTx. Secondly, this study was retrospective rather than prospective, introducing potential bias. Thirdly, the sample size was small, limiting the generalizability of the findings. Fourthly, we were unable to analyze the safety profiles of the third-line CTx in sufficient number of patients. Further analysis of the safety profile is needed. To overcome these limitations, a prospective analysis of other cohorts is currently underway to confirm the reproducibility of the results. Finally, the absence of safety evaluation posed a challenge, as assessing safety is crucial in tertiary care. However, this aspect could not be addressed within the scope of this study. To prove the efficacy of third-line CTx in patients with UPC, conducting a prospective randomized trial with an increased sample size is necessary to evaluate the efficacy and safety of this treatment. Additionally, although this study contributes valuable data, comparing third-line CTx outcomes to those of BSC remains challenging due to the complexities of tertiary care. Further studies are warranted to generate robust evidence supporting the findings of this study.

In conclusion, third-line CTx should be actively considered for patients with UPC, as those who can tolerate the treatment may experience prolonged survival. Furthermore, the appropriate regimen will need to be verified in future prospective comparative trials.

## Data Availability

The raw data supporting the conclusions of this article will be made available by the authors, without undue reservation.

## Supplementary material

Supplementary Figure 1 Progression-free survival following second-line chemotherapy. BSC, best supportive care; HR, hazard ratio; CI, confidence interval.
