# Supplementary figures and images for: Efficacy of third-line chemotherapy following nanoliposomal irinotecan combined with fluorouracil and folinic acid as second-line treatment for unresectable pancreatic cancer
Source: Front Oncol. 2025 Jul 17;15:1626689. doi: 10.3389/fonc.2025.1626689 (PMC12310504; doi:10.3389/fonc.2025.1626689)

## Slide 1
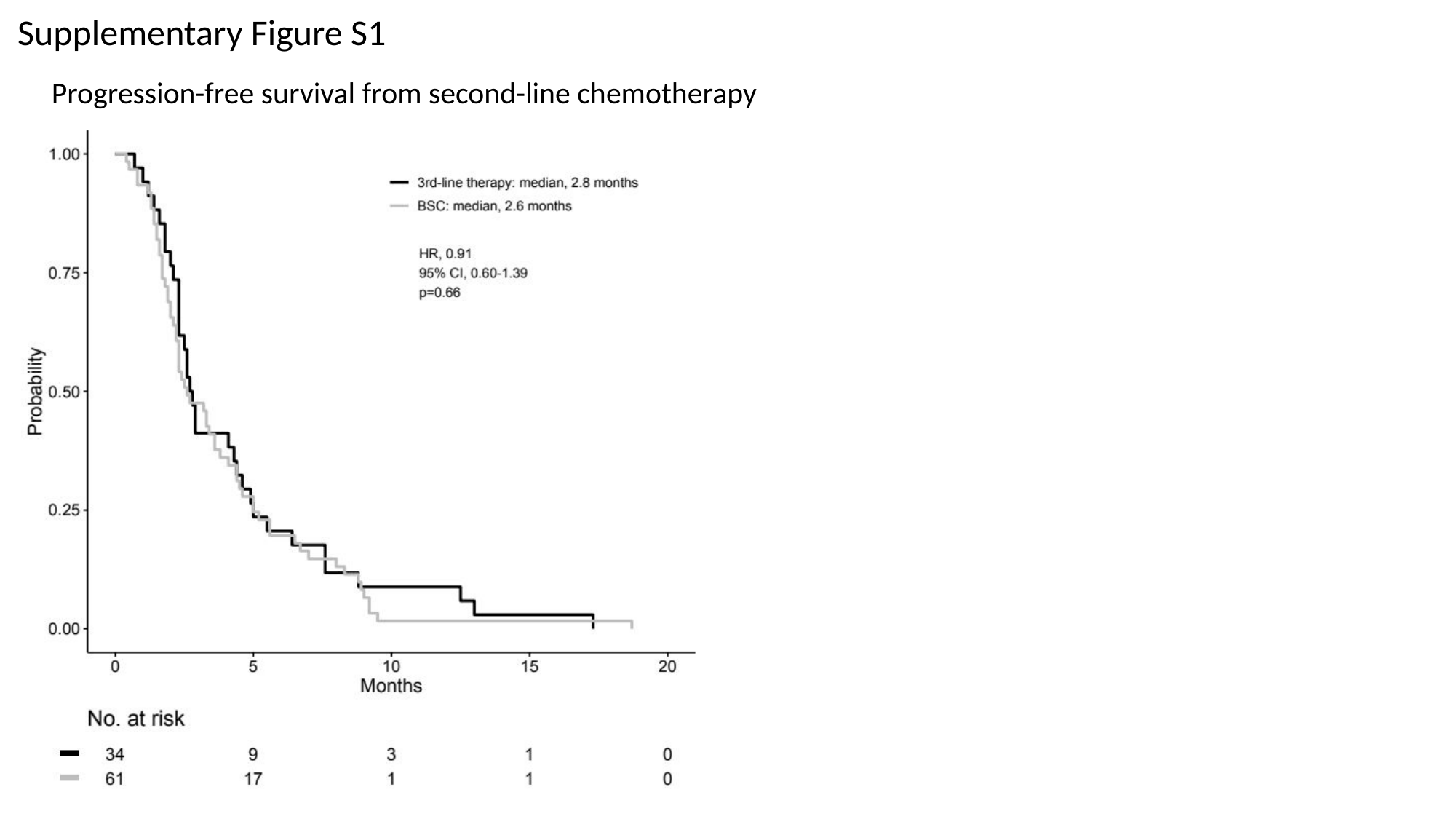

Supplementary Figure S1
Progression-free survival from second-line chemotherapy

Supplement: Supplementary Figure 1 — Progression-free survival following second-line chemotherapy. BSC, best supportive care; HR, hazard ratio; CI, confidence interval. [file Presentation1.pptx]
